# Intraindividual variability in behavior shapes fitness landscapes

**DOI:** 10.1002/ece3.6099

**Published:** 2020-02-15

**Authors:** Toshinori Okuyama

**Affiliations:** ^1^ Department of Entomology National Taiwan University Taipei Taiwan

**Keywords:** individual‐based model, intraindividual variability, within‐individual variation

## Abstract

Although intraindividual variability (IIV) in behavior is fundamental to ecological dynamics, the factors that contribute to the expression of IIV are poorly understood. Using an individual‐based model, this study examined the effects of stochasticity on the evolution of IIV represented by the residual variability of behavior. The model describes a population of prey with nonoverlapping generations, in which prey take refuge upon encountering a predator. The strategy of a prey is characterized by the mean and IIV (i.e., standard deviation) of hiding duration. Prey with no IIV will spend the same duration hiding in a refuge at each predator encounter, while prey with IIV will have variable hiding durations among encounters. For the sources of stochasticity, within‐generation stochasticity (represented by random predator encounters) and between‐generation stochasticity (represented by random resource availability) were considered. Analysis of the model indicates that individuals with high levels of IIV are maintained in a population in the presence of between‐generation stochasticity even though the optimal strategy in each generation is a strategy with no IIV, regardless of the presence or absence of within‐generation stochasticity. This contradictory pattern emerges because the mean behavioral trait and IIV do not independently influence fitness (e.g., the sign of the selection gradient with respect to IIV depends on the mean trait). Consequently, even when evolution eventually leads toward a strategy with no IIV (i.e., the optimal strategy), greater IIV may be transiently selected. Between‐generation stochasticity consistently imposes such transient selection and maintain high levels of IIV in a population.

## Graphical abstract

Fitness contours based on the mean (horizontal axis) and intraindividual variability (IIV) (vertical axis) in behavior are tilted and create positive selection gradients with respect to IIV even though the optimal strategy is a strategy with no IIV. When populations consist of individuals with nonoptimal traits (e.g., [population 1] mean = 15 & IIV = 0, [population 2] mean = 20 & IIV = 0, [population 3] mean = 25 & IIV = 0), evolution takes long detours and transiently increase IIV, whose trajectories are depicted by the black lines.

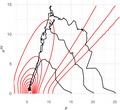

## 1 INTRODUCTION

Intraindividual variability (IIV) in behavior, which may be interpreted as unpredictability in behavior, is a trait of individuals. At its simplest representation, a single individual can behave randomly, or unpredictably, under the same conditions. Precisely speaking, it is impossible to observe the same individual repeatedly under the same conditions, because some attributes of the individual, such as age and experience, will inevitably change. However, even after accounting for those changes, the behavior of an individual cannot be precisely predicted due to IIV (Stamps, Briffa, & Biro, 2012). IIV can be a source of within‐species variability and can influence a range of ecological dynamics (Bolnick et al., 2011; Des Roches et al., 2018; Okuyama, 2008). Understanding the expression of IIV is therefore important, even when IIV is not the main focus of interest.

Patterns observed in the expressions of IIV suggest that IIV has important fitness consequences. For example, some individuals consistently express greater IIV than others (Biro & Adriaenssens, 2013; Briffa, 2013; He, Pagani‐Nunez, Chevallier, & Barnett, 2017; Highcock & Carter, 2014; Stamps et al., 2012). The expression of IIV may not be constant within an individual (e.g., an individual may adjust the level of IIV across contexts) (Jolles, Briggs, Araya‐Ajoy, & Boogert, 2019; Mathot & Dingemanse, 2014), which implies IIV can be expressed as behavioral plasticity. When IIV is a heritable trait (Henriksen, 2019), these observed patterns are results of natural selection on IIV. However, optimal behavior models typically consider only the mean expression of behavior (Charnov, 1976; Cooper & Frederick, 2007), and relationships between behavioral IIV and fitness are largely unknown.

Stochasticity is a possible factor that influences the relationship between IIV and fitness. One source of stochasticity is within‐generation stochasticity that is the stochasticity realized within a generation (e.g., within the life span of individuals). Such within‐generation stochasticity and behavioral stochasticity (IIV) may interact and influence fitness. Another source of stochasticity is between‐generation stochasticity. A good behavioral strategy in the current generation may be a poor strategy in the offspring's generation when environmental conditions change over generations (i.e., the optimal strategy depends on environmental conditions). Previous studies examined how environmental changes influence the evolution of adaptive traits and subsequent population dynamics (Abrams, 2001, 2003; Abrams & Matsuda, 1997), but IIV was not considered in them.

The study used an individual‐based model to examine the effects of the two types of stochasticity on IIV. For the within‐generation and between‐generation stochasticity, predator encounters and resource availability, respectively, were considered. The behavior considered is a hiding behavior of prey. Various prey species hide in a refuge such as a burrow or shell when they encounter predators (Everett & Ruiz, 1993; Kramer & Bonenfant, 1997; Martin, Lopez, & Cooper, 2003; Mima, Wada, & Goshima, 2003). When a prey experiences multiple predator encounters, the duration of hiding may vary with each encounter, which is an expression of IIV.

## 2 THE MODEL

The model discussed in this study is similar to exiting prey refuge models (Cooper & Frederick, 2007; Martin & Lopez, 1999) in which the optimal strategy is derived by balancing the cost and benefit of hiding (described below). The model is a discrete generation individual‐based model in which prey can live for a generation, and all the individuals in the following generation are the offspring of the current generation individuals. Reproductions take place at the end of each generation by prey that survived till that time. The model assumes IIV is a heritable trait. Because successful individuals will reproduce more offspring that share the traits of successful individuals, simulations will impose natural selection on the behavior. The effects of natural selection on both the mean and variability (IIV) of the behavior can be examined by the distributions of behavioral traits over generations.

### 2.1 Intraindividual variation

The individual‐based model describes a situation in which prey survive predator encounters and are subsequently able to reproduce. When a prey individual encounters a predator, it hides in a refuge for a length of time which follows a gamma distribution with mean *µ_i_* and standard deviation $\sigma_{i}^{\text{IIV}}$, where *µ_i_* and $\sigma_{i}^{\text{IIV}}$ are traits unique to the individual *i* (*i* = 1, 2, …, *K* when there are *K* individuals in the population). A gamma distribution is used because hiding duration takes non‐negative continuous values. When a prey experiences *p* predator encounters, hiding durations vary for each encounter when $\sigma_{i}^{\text{IIV}}>0$. In other words, $\sigma_{i}^{\text{IIV}}$ represents IIV, and $\sigma_{i}^{\text{IIV}}$ $>\sigma_{j}^{\text{IIV}}$ indicates that individual *i* exhibits greater IIV than individual *j*. By convention, when $\sigma_{i}^{\text{IIV}}=0$, individual *i* will always spend a hiding duration of *µ_i_* for each predator encounter, exhibiting no IIV. It is assumed that the strategy is fixed, and a prey does not flexibly adjust *µ_i_* and $\sigma_{i}^{\text{IIV}}$ in its lifetime. $\sigma_{i}^{\text{IIV}}$ may be interpreted as the residual variability of behavior, rather than behavioral plasticity.

### 2.2 Survival and reproduction

Hiding has both benefits and costs. As the duration of hiding increases, the probability of survival after an encounter with a predator increases. However, hiding reduces the time available for other activities, such as forging for food or other resources. For a prey individual which has remained in a refuge for a duration *t*, its survival probability *s*(*t*) is described by.

(1) $$\text{logit}\left(s\left(t\right)\right)=a+bt$$

in which *a* and *b* are the parameters that determine the relationship. For example, *b* > 0 indicates that the longer a prey remains in a refuge, the greater the possibility of survival.

To reproduce, the prey must survive until the end of the season by successfully escaping *p* predators. For example, if a prey experiences three predator encounters (*p* = 3), it must survive all three encounters. The total hiding duration *t*_tot_ of the prey is the sum of the three hiding durations, such that *t*_tot_ = *t*_1_ + *t*_2_ + *t*_3_ where *t_i_* is the hiding duration from *i*th predator encounter. For a prey that has survived all encounters with *t*_tot_, its reproductive potential *r*(*t*_tot_) is also modeled as the logit function.

(2) $$\text{logit}\left(r\left(t_{\text{tot}}\right)\right)=\alpha -\beta t_{\text{tot}}$$

where *α* and *β* are the parameters that determine the relationship. In Eq. 2, *β* > 0 indicates the cost to reproduction of hiding. *β* is negatively correlated with resource availability, and hiding duration (*t*_tot_) has a greater cost when resource level is low. Because *r*(*t*_tot_) is not a probability, the use of the logit function is not necessary, but the same functional form is used for the benefit (Eq. 1) and cost (Eq. 2) of hiding, simply for consistency. Using different functions such as *r*(*t*_tot_) = *αe* ^−^ *^βt^*_tot_ can also give equivalent results, and the results are not sensitive to the functional form of *r*(*t*_tot_). The relationship between reproductive potential *r*(*t*_tot_) and actual reproduction is described below (Eq. 3).

**Figure 1:**
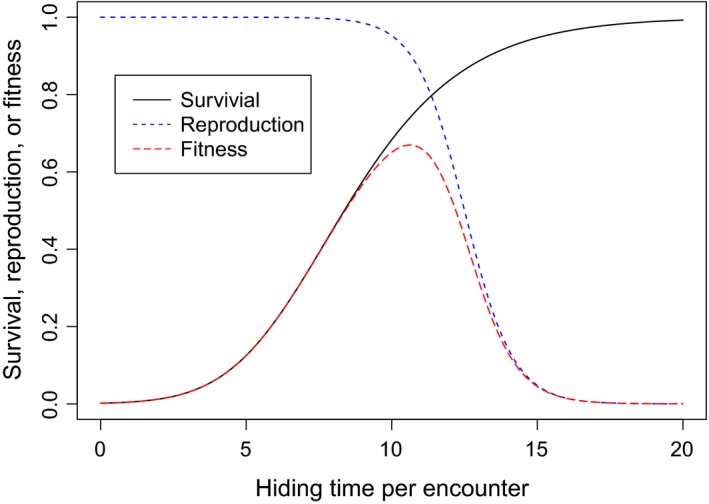
Effects of hiding time on survival, reproduction, and fitness when prey encounter three predators (*p* = 3). Survival is determined by Eq. 1 (i.e., *s*(*t*)*^p^*), and reproduction is determined by Eq. 2 (i.e. *b*(*pt*)). The resulting fitness is the product of the two, *s*(*t*)*^p^b*(*pt*). Parameters: *a* = −2, *b* = 0.4, *α* = 15, β = 0.4

The optimal hiding duration is determined by the benefit of hiding, *s*(*t*), and the cost of hiding, 1−*r*(*t*_tot_); both increase with hiding time. For a specific case, the optimal hiding duration can be easily derived. Figure 1 shows the result for a situation where prey encounters three predators (*p* = 3) and no IIV (hiding duration for each encounter is *t*; *t*_tot_ = *pt*). The optimal hiding duration is *t* that maximizes the fitness function *s*(*t*)*^p^r*(*pt*).

#### 2.2.1 Performance of individuals

The behavioral strategy of *i*th prey in a population is represented by (*µ_i_* and $\sigma_{i}^{\text{IIV}}$). In each generation, the following steps are taken for each prey individual to determine its reproductive potential: (1) determine the number of predator encounters, *p*; (2) generate *p* hiding durations (i.e., *t*_1_, …, *t_p_*) from a gamma distribution with mean *µ_i_* and standard deviation $\sigma_{i}^{\text{IIV}}$; (3) determine whether the prey survives *p* encounters in which each encounter is a Bernoulli process in which the survival probability is determined by Eq. 1; and (4) if the prey survives, calculate its reproductive potential based on Eq. 2. If the prey dies in a predator encounter, its reproductive potential is 0. The distributional assumption for *p*, which represents within‐generation stochasticity, is described below.

#### 2.2.2 Fitness landscapes

Because the simulation (section 2.2.1) is a stochastic simulation, the resulting value for the reproductive potential for the same strategy (i.e., a particular combination of *μ* and *σ* ^IIV^) is variable each time it is simulated. The expected reproductive potential for a specific trait can be obtained by running the simulation many times and taking the average (1 million simulation runs were used to compute an average). Because a behavioral strategy consists of two components (*μ* and *σ* ^IIV^), computing the expected reproductive potential for various combinations of *μ* and *σ* ^IIV^ will describe the relationship between behavioral strategies and fitness on a two‐dimensional space, which is referred as fitness landscape. Because prey lives only one generation, the optimal strategy is the strategy that results in the highest expected reproductive potential in the simulation (section 2.2.1). Between‐generation stochasticity does not affect the optimal strategy.

### 2.3 Evolution

The model assumes that the carrying capacity of the environment is *K*, and there is *K* prey in each generation at the beginning of the simulation. For example, the combined reproduction of all members of the population is more than *K* offspring, assuming that at least one prey survives to the end of the generation, but in each generation, a random set of *K* offspring will survive to begin the next generation. The number of offspring from prey individual *i* that survives to begin the next generation is a random variable *X_i_* such that *X*_1_ + *X*_2_ + … + *X_K_* = *K*. In particular, *X* = (*X*_1_, *X*_2_, …, *X_K_*) follows a multinomial distribution,

(3) X∼Multinomial(K,π)

where *π* = (*π*_1_, *π*_2_, …, *π_K_*) is the probability vector, and $\pi_{i}=r_{i}/\sum_{j=1}^{K}r_{j}\left(i=1,\;\ldots ,\;K\right)$. Therefore, prey with higher reproductive potentials are expected to leave more offspring.

Each offspring produced by a parent prey with a strategy (*μ* and *σ* ^IIV^) will inherit the traits of the parent. An offspring's mean trait is *μ* + *q*_µ_ and IIV trait is *σ* ^IIV^ + *q*_σ_ where *q*_µ_ and *q*_σ_ are random numbers generated from a normal distribution with mean 0 and standard deviations *s*_µ_ and *s*_σ_, respectively. *s*_µ_ and *s*_σ_ are regarded as surrogates of heritability, although they are negatively correlated with heritability (e.g., *s*_µ_ = 0 and *s*_σ_ = 0 when a parent and its offspring all have the same traits, and heritability decreases as *s*_µ_ and *s*_σ_ increase). When a generated trait value becomes negative (e.g., *σ* ^IIV^ + *q*_σ_ < 0), the value is set to 0, because both the mean and standard deviation of the hiding time cannot be negative. This study assumed small values of *s*_µ_ and *s*_σ_ (Table 1), because a large value of *s*_σ_, in particular, by definition increases variation in *σ* ^IIV^ without any other mechanisms. Because of the inheritance, traits associated with successful individuals will spread in the population. By tracking the traits of individuals in the population over generations, it is possible to examine how selection influences the combination of *μ* and *σ* ^IIV^. The traits of the individuals after 1,000 generations were examined as the outcome of evolution. Unless otherwise stated, the initial population consisted of all individuals with *μ* = 1 and σ^IIV^ = 0.

**Table 1:** Parameter definitions and values. When a symbol represents a realization of a random variable, its distribution is shown. For example, *t* follows a gamma distribution with mean *μ* and standard deviation (SD) *σ* ^IIV^. When SD is 0, it is constant regardless of the distribution

	Symbol	Distribution	Mean	*SD*
Hiding time (per encounter)	*t*	gamma	*μ*	*σ* ^IIV^
Survival (slope)	*a*	constant	−2	0
Survival (intercept)	*b*	constant	0.4	0
Reproduction (slope)	*α*	constant	15	0
Reproduction (intercept)	β	gamma	0.4	0 or 0.3
Number of predator encounters	*p*	negative binomial	3	0 or 4
Surrogate of heritability for *μ*	*q*_μ_	normal	0	0.5
Surrogate of heritability for *σ* ^IIV^	*q*_σ_	normal	0	0.5
Carrying capacity	*K*	constant	1,000	0

### 2.4 Parameters and stochasticity

The model described above is complete when the parameter values are determined (Table 1). The survival parameters (*a* and *b*) were set so that when a prey does not hide (*t* = 0), the probability of survival is approximately 0.12. One unit of hiding duration increases the odds of survival by 1.5 times. The reproductive parameters (*α* and β) were set such that the reproductive potential is approximately 1 for prey that did not waste any time on hiding, given that they survive, and by setting β = *b*, the cost (β) and benefit (*b*) of hiding were on a similar scale in an average environment. The expected number of predator encounters is not independent of the survival parameters (*a* and *b*) in the model. That is, a high expected number of encounters with a high per‐encounter survival rate, and a low expected number of encounters with a low per‐encounter survival rate will give similar outcomes. For a given set of *a* and *b*, as the number of predator encounters increase, eventually no individuals can survive and reproduce and the population collapses. When the number of encounters is too few, there is very weak selection on hiding traits. The number of encounters was set to balance these factors.

To examine the effect of within‐generation stochasticity, the expected number of predator encounters was set at 3, and results from two levels of variability (i.e., standard deviation is 0 and 4) were compared. Zero standard deviation indicates a constant (i.e., all individuals encounter 3 predators), and the stochastic predator encounters were simulated by a negative binomial distribution. Between‐generation stochasticity was represented by variability in β (in Eq. 2) among generations. In each generation, the value of β was generated from a gamma distribution with a standard deviation of 0.3 (Table 1). Greater stochasticity will enhance patterns that will be shown in the *Results*.

## 3 RESULTS

### 3.1 Effects of within‐generation stochasticity

**Figure 2:**
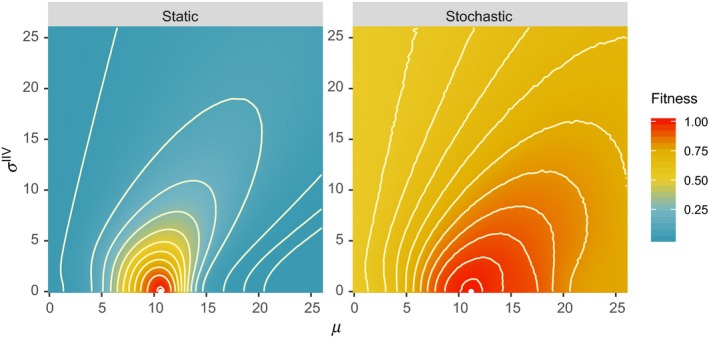
Fitness landscapes under a static environment and stochastic environment. In the static environment, the number of predator encounters is fixed at 3. In the stochastic environment, the average and standard deviation of the number of predator encounters, respectively, are 3 and 4 (simulated by a negative binomial distribution). The expected values of reproductive potential are scaled such that the maximum reproductive potential is 1. In each plot, the point at the center of contours corresponds with the maximum fitness: (static: *µ* = 10.66, σ^IIV^ = 0) and (stochastic: *µ* = 11.18, σ^IIV^ = 0)

Stochastic predator encounter does not qualitatively change the fitness landscape (Figure 2). Both in the static condition (i.e., all prey encounter three predators) and in the stochastic environment (i.e., the number of predator encounters is variable), the optimal strategy is associated with a strategy with no IIV, σ^IIV^ = 0. The main difference is that the fitness landscape becomes flat (i.e., a wide range of strategies perform equivalently well with respect to the optimal strategy) in the stochastic environment.

**Figure 3:**
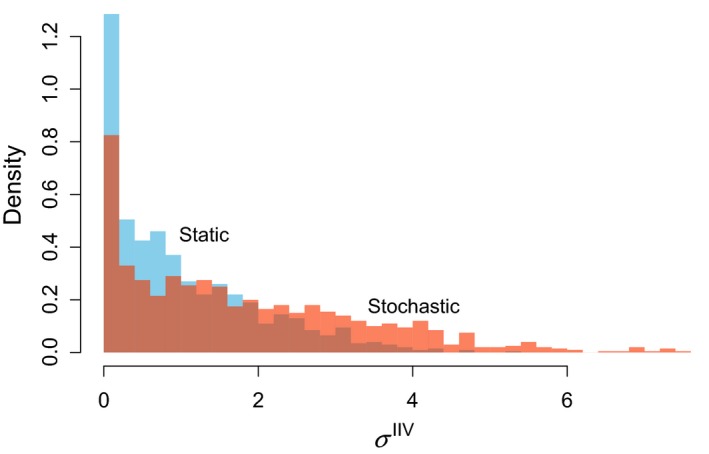
Histograms of σ^IIV^ after 1,000 generations. In the static environment, the number of predator encounters is fixed at 3. In the stochastic environment, the average and standard deviation of the number of predator encounters, respectively, are 3 and 4 (simulated by a negative binomial distribution)

After 1,000 generations of the evolutionary simulation (section 2.3), the mode of the distribution of σ^IIV^ is 0 for both cases (Figure 3). Movies of these simulations are provided as Supporting Information, which shows how individual traits evolve in all 1,000 generations. Due to the imperfect heritability and stochastic processes in the model, many individuals exhibit IIV (σ^IIV^ > 0). Higher levels of IIV are maintained under the stochastic environment than in the static environment (Figure 3). This result directly comes from the flat fitness landscape (Figure 2). When predator encounter is stochastic, some prey does not encounter any predators. Consequently, those lucky prey will leave many offspring regardless of their strategies. Similarly, prey that encounters unusually many predators (by chance) are likely die and leave no offspring regardless of their strategies. Consequently, the chance events (rather than strategy) becomes a dominant factor in determining fitness, making the fitness landscape flat. In both cases, expressions of IIV (e.g., individuals with σ^IIV^ > 0 in Figure 3) can be interpreted as spill‐overs from the optimal strategy (σ^IIV^ = 0) due to imperfect heritability and chance events. This study does not focus on this trivial mechanism (weak selection), and the following results will assume the static number of predator encounters to examine the evolution of IIV under an assumption selection strongly acts on hiding strategies.

### 3.2 Effects of between‐generation stochasticity

**Figure 4:**
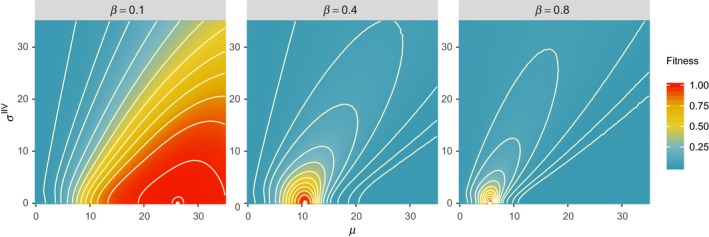
Fitness landscapes under three different values of β. The three values approximately relate to the 10th percentile (β = 0.1), mean (β = 0.4), and 90th percentile (β = 0.8) of β in the stochastic environment. The expected values of reproductive potential are scaled such that the maximum reproductive potential is 1. In each plot, the point at the center of contours corresponds with the maximum fitness: (β = 0.1: *µ* = 26.25, σ^IIV^ = 0), (β = 0.4: *µ* = 10.5, σ^IIV^ = 0), and (β = 0.8: *µ* = 5.6, σ^IIV^ = 0)

The fitness landscapes are shown for three values of β: 0.1, 0.4, and 0.8 (Figure 4). The three values approximately relate to the 10th percentile (β = 0.1), mean (β = 0.4), and 90th percentile (β = 0.8) of β in the stochastic environment. A general pattern is that as the environmental condition becomes worse and β increases, the optimal expected hiding duration *μ* decreases. This result is intuitive, because in an unfavorable environment, more activity time is needed to secure resources for reproduction. Two important results are as follows: the optimal strategy is associated with σ^IIV^ = 0 regardless of the environmental condition (β); and the effects of *μ* and σ^IIV^ on fitness are not independent so that the fitness contour is tilted. When β changes over generations, fitness landscape will also change accordingly, creating dynamic fitness landscape.

**Figure 5:**
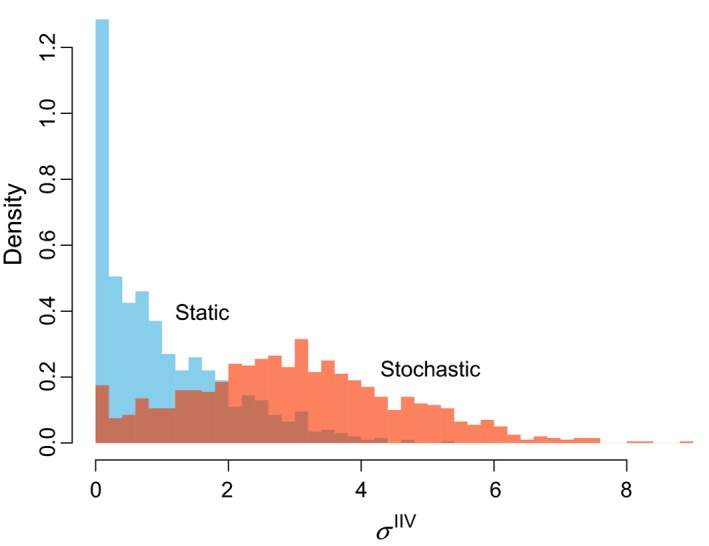
Histograms of σ^IIV^ after 1,000 generations. In the static environment, β is fixed at 0.4. In the stochastic environment, β was generated from a gamma distribution with mean 0.4 and standard deviation 0.3 in each generation

After 1,000 generations, much greater levels of IIV are maintained in the population under the stochastic environment than in the static environment (Figure 5). The distribution of σ^IIV^ randomly fluctuates in the stochastic environment. Movies of these simulations are provided as Supporting Information, which shows how individual traits evolve in all 1,000 generations. In the absence of environmental stochasticity (β = 0.4 for all generations), the distribution of traits converges and fluctuate little. In the stochastic environment, the mode of the distribution of σ^IIV^ is not necessarily 0 (Figure 5), which is different from the case for within‐generation stochasticity (Figure 3) in which the variability comes from spill‐overs from the optimal strategy (σ^IIV^ = 0) due to weak selection. In addition, the movie (Supporting Information) show that greater σ^IIV^ is periodically selected in the presence of the between‐generation stochasticity. Because the optimal strategy is associated with σ^IIV^ = 0 in each generation (Figure 4), selection for greater σ^IIV^ deserves an explanation.

### 3.3 Evolutionary trajectory

**Figure 6:**
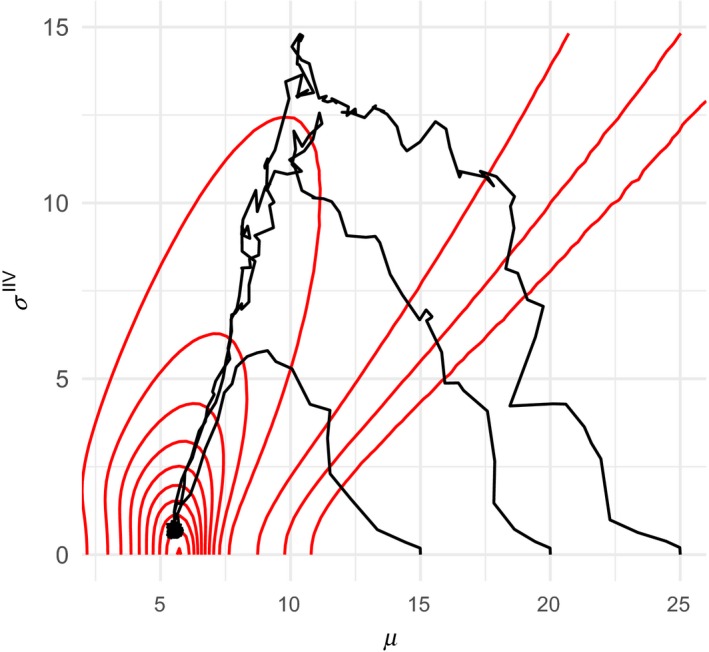
Evolutionary trajectories of the average hiding strategy under a harsh environment (β = 0.8). The contour shows the corresponding fitness landscape that is the same as the one shown in Figure 4. Results of simulations using three different initial conditions are shown. The initial population consisted of individuals with $\sigma_{i}^{\text{IIV}}=0$ for all *i*, and *µ_i_* = 15, *µ_i_* = 20, and *µ_i_* = 25 for all *i*. Average trait values were tracked for 1,000 generations. In all three cases, although the trajectories take a detour, they all eventually reached the same optimal solution

When the strategies of individuals in a population are suboptimal, natural selection will eventually lead to the optimal strategy over generations. However, the average strategy of the population will not approach the optimal strategy by its shortest path. The gradient of a fitness landscape (e.g., contour in Figure 4) determines the direction of evolution. This can be clearly illustrated by a condition where the environmental conditions are constantly bad (e.g., β = 0.8 for all generations), and the population consists of individuals whose strategies are all far from the optimal strategy. Figure 6 shows the evolutionary trajectories from three populations with different initial conditions: All individuals have (*µ* = 15, *σ* ^IIV^ = 0), (*µ* = 20, *σ* ^IIV^ = 0), or (*µ* = 25, *σ* ^IIV^ = 0). Regardless of the initial conditions, they all eventually reach the same strategy, but the evolutionary trajectories take long detour. Because the fitness contour is generally tilted to the right (Figure 4), when environmental condition becomes worse (e.g., β changes from 0.1 to 0.8), selection tends to favor individuals with greater *σ* ^IIV^. In the presence of between‐generation stochasticity, when the environmental condition is worsened, individuals with high IIV may be transiently selected even when the optimal strategy in that condition is associated with *σ* ^IIV^ = 0, which maintains individuals with high IIV in the population. Figure 6 shows average traits over generations, and the corresponding movie that shows the traits of all individuals when the initial population is characterized by the strategy (*µ* = 25, *σ* ^IIV^ = 0) is provided as Supporting Information.

## 4 DISCUSSION

This study examined the effects of two types of stochasticity on the evolution of IIV. First, within‐generation stochasticity has little effect on the optimal strategy but still can influence the evolution of IIV by weakening selection. Second, between‐generation stochasticity can maintain high levels of IIV when two conditions are satisfied: (a) The optimal strategy depends on the environment and changes over generations; and (b) the mean and IIV of behavior influencing fitness in a nonindependent manner. Although a specific model was considered in the current study, these two conditions hold in a variety of situations.

The first condition, the dynamic optimal strategy, is commonly acknowledged, and has been intensively studied. In any optimality models, the optimal strategy will change according to the environmental variables considered in the models. For example, optimal patch residence time in the marginal value theorem can change, for example, with patch quality (Charnov, 1976). Optimal prey choice depends upon the density of profitable prey species (Pulliam, 1974). As such, this condition is rather trivial and is likely satisfied in various ecological scenarios.

**Figure 7:**
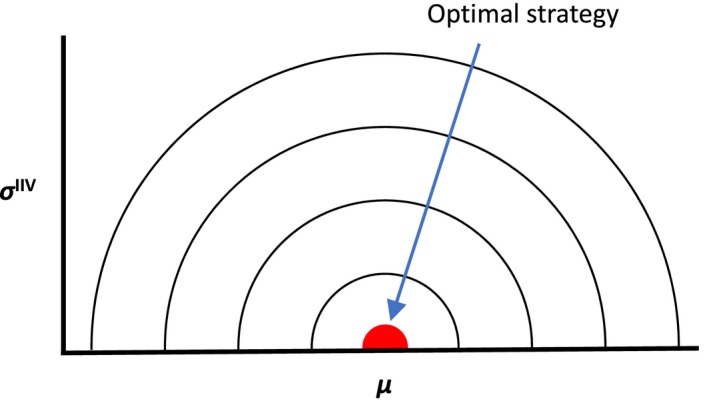
An example of fitness contours when μ and σ^IIV^ do not interact. For any suboptimal strategies with σ^IIV^ > 0, the fitness gradient with respect to σ^IIV^ is negative (smaller σ^IIV^ is selected)

The second condition, that mean behavioral expression and IIV do not independently influence fitness, is also likely to be valid in many situations. To express the idea clearly, an example of fitness contour when *µ* and *σ* ^IIV^ do not interact is shown in Figure 7. The reason why *µ* and *σ* ^IIV^ interact can be illustrated with a simple generic example. Suppose that the relationship between behavior *x* and fitness *w* can be represented by *w*(*x*) = −(*x*−1)^2^ + 1 (when 0 ≤ x ≤ 2) and *w*(*x*) = 0 (when *x* > 2). A specific *w*(*x*) is used here but any unimodal functions (e.g., Figure 1) will work in the same way. The optimal strategy for this example is (*µ* = 1, *σ* ^IIV^ = 0), and any strategies with (*µ* > 2, *σ* ^IIV^ = 0) have 0 fitness. However, strategies (*µ* > 2, *σ* ^IIV^ > 0) (e.g., *µ* = 2.1, *σ* ^IIV^ = 1) have a positive expected fitness because IIV (*σ* ^IIV^ > 0) can produce *x* between 0 and 2 by chance. Therefore, the effect of *σ* ^IIV^ on fitness is not independent of *µ*, and the fitness contours will not be true circles as in Figure 7. Although unlikely, when the second condition is not satisfied, transient selection for greater *σ* ^IIV^ will not occur even when the first condition is satisfied because the selection gradient with respect to *σ* ^IIV^ is always directed toward *σ* ^IIV^ = 0.

Theoretical studies that examine the evolution of traits in dynamical systems typically assume that a trait evolves to the direction of the selection gradient (e.g., Abrams, Matsuda, & Harada, 1993), which is the same as the result of this study in which evolution tracks the fitness gradient (Figure 6). However, in those studies, only the gradient with respect to mean trait ∂w/∂μ is considered, and the gradient with respect to variability, $\partial w/\partial \sigma^{IIV}$ is not considered. This assumption (ignoring IIV) may be valid when inflexible traits (e.g., some morphological traits) are considered but may not be appropriate for traits with IIV (e.g., behavior). An important conclusion of this study is that even when the optimal strategy is associated with *σ* ^IIV^ = 0, the selection gradient with respect to IIV, $\partial w/\partial \sigma^{\text{IIV}}$, cannot be neglected.

Weak selection can have substantial influence on IIV traits of individuals in a population (Figure 3). When selection is weak, random effects can overrule selection. In other words, fitness is largely influenced by luck rather than strategies. Although trivial, it does not necessarily mean it is unimportant. In case of hiding duration, there may be indeed a substantial proportion of individuals that may never encounter predators in natural conditions. Or predation risk per encounter may be generally very low regardless of the strategy. When we focus on the optimal strategy, we can still find a unique optimal strategy no matter how flat a fitness landscape may be (Figure 2), but the strength of selection must be carefully examined in each case.

One immediate prediction of the model presented here is that higher levels of IIV are expected in populations that experience greater environmental variability regardless of within‐generation variability (i.e., weak selection) or between‐generation (i.e., transient selection). To better examine the evolutionary mechanism presented in this study, we must be able to quantify fitness landscapes. We currently know little about how IIV influences fitness, and less is known about how *µ* and *σ* ^IIV^ jointly influence fitness. Figures 2 and 4 show that IIV can have both positive and negative effects, depending on mean expression, and thus a particular experimental result that simply shows a positive or negative effect of IIV may be incomplete. Similarly, a study that only focused on IIV may give misleading conclusions when the mean expressions are not accounted for in the experimental design and data analysis. A simultaneous consideration of both mean and IIV may provide a better understanding of the effect of IIV on individual fitness, as well as how it is maintained in populations.

## CONFLICT OF INTEREST

None declared.

## AUTHOR CONTRIBUTION

TO performed all work presented in this study.

## Data Availability

This study is not based on data.

## References

[ece36099-bib-0001] Abrams, P. A. (2001). Modelling the adaptive dynamics of traits involved in inter‐ and intraspecific interactions: An assessment of three methods. Ecology Letters, 4, 166–175. 10.1046/j.1461-0248.2001.00199.x

[ece36099-bib-0002] Abrams, P. A. (2003). Can adaptive evolution or behaviour lead to diversification of traits determining a trade‐off between foraging gain and predation risk? Evolutionary Ecology Research, 5, 653–670.

[ece36099-bib-0003] Abrams, P. A. , & Matsuda, H. (1997). Prey adaptation as a cause of predator–prey cycles. Evolution, 51, 1742–1750. 10.1111/j.1558-5646.1997.tb05098.x 28565102

[ece36099-bib-0004] Abrams, P. A. , Matsuda, H. , & Harada, Y. (1993). Evolutionarily unstable fitness maxima and stable fitness minima of continuous traits. Evolutionary Ecology, 7, 465–487. 10.1007/BF01237642

[ece36099-bib-0005] Biro, P. A. , & Adriaenssens, B. (2013). Predictability as a personality trait: Consistent differences in intraindividual behavioral variation. American Naturalist, 182, 621–629. 10.1086/673213 24107369

[ece36099-bib-0006] Bolnick, D. I. , Amarasekare, P. , Araújo, M. S. , Bürger, R. , Levine, J. M. , Novak, M. , … Vasseur, D. A. (2011). Why intraspecific trait variation matters in community ecology. Trends in Ecology & Evolution, 26, 183–192. 10.1016/j.tree.2011.01.009 21367482 PMC3088364

[ece36099-bib-0007] Briffa, M. (2013). Plastic proteans: Reduced predictability in the face of predation risk in hermit crabs. Biology Letters, 9 10.1098/rsbl.2013.0592 PMC3971704 23985348

[ece36099-bib-0008] Charnov, E. L. (1976). Optimal foraging, the marginal value theorem. Theoretical Population Biology, 9, 129–136. 10.1016/0040-5809(76)90040-X 1273796

[ece36099-bib-0009] Cooper, W. E. , & Frederick, W. G. (2007). Optimal time to emerge from refuge. Biological Journal of the Linnean Society, 91, 375–382. 10.1111/j.1095-8312.2007.00802.x

[ece36099-bib-0010] Des Roches, S. , Post, D. M. , Turley, N. E. , Bailey, J. K. , Hendry, A. P. , Kinnison, M. T. , … Palkovacs, E. P. (2018). The ecological importance of intraspecific variation. Nature Ecology and Evolution, 2, 57–64. 10.1038/s41559-017-0402-5 29203921

[ece36099-bib-0011] Everett, R. A. , & Ruiz, G. M. (1993). Coarse woody debris as a refuge from predation in aquatic communities — an experimental test. Oecologia, 93, 475–486. 10.1007/BF00328954 28313814

[ece36099-bib-0012] He, R. C. , Pagani‐Nunez, E. , Chevallier, C. , & Barnett, C. R. A. (2017). To be so bold: Boldness is repeatable and related to within individual behavioural variability in North Island robins. Behavioural Processes, 140, 144–149. 10.1016/j.beproc.2017.04.014 28454917

[ece36099-bib-0013] Henriksen, R. et al (2019). Intra‐individual behavioural variability: A trait under genetic control. bioRxiv, 10.1101/795864 PMC7663371 33138119

[ece36099-bib-0014] Highcock, L. , & Carter, A. J. (2014). Intraindividual variability of boldness is repeatable across contexts in a wild lizard. PLoS ONE, 9, e95179 10.1371/journal.pone.0095179 24733271 PMC3986370

[ece36099-bib-0015] Jolles, J. W. , Briggs, H. D. , Araya‐Ajoy, Y. G. , & Boogert, N. J. (2019). Personality, plasticity and predictability in sticklebacks: Bold fish are less plastic and more predictable than shy fish. Animal Behaviour, 154, 193–202. 10.1016/j.anbehav.2019.06.022

[ece36099-bib-0016] Kramer, D. L. , & Bonenfant, M. (1997). Direction of predator approach and the decision to flee to a refuge. Animal Behaviour, 54, 289–295. 10.1006/anbe.1996.0360 9268459

[ece36099-bib-0017] Martin, J. , & Lopez, P. (1999). When to come out from a refuge: Risk‐sensitive and state‐dependent decisions in an alpine lizard. Behavioral Ecology, 10, 487–492. 10.1093/beheco/10.5.487

[ece36099-bib-0018] Martin, J. , Lopez, P. , & Cooper, W. E. (2003). When to come out from a refuge: Balancing predation risk and foraging opportunities in an alpine lizard. Ethology, 109, 77–87. 10.1046/j.1439-0310.2003.00855.x

[ece36099-bib-0019] Mathot, K. J. , & Dingemanse, N. J. (2014). Plasticity and personality In Martin L. B., Ghalambor C. K., & Woods H. A. (Eds.), Integrative organismal biology (pp. 55–69). Hoboken, NJ: Wiley.

[ece36099-bib-0020] Mima, A. , Wada, S. , & Goshima, S. (2003). Antipredator defence of the hermit crab *Pagurus filholi* induced by predatory crabs. Oikos, 102, 104–110. 10.1034/j.1600-0706.2003.12361.x

[ece36099-bib-0021] Okuyama, T. (2008). Individual behavioral variation in predator–prey models. Ecological Research, 23, 665–671. 10.1007/s11284-007-0425-5

[ece36099-bib-0022] Pulliam, H. R. (1974). On the theory of optimal diets. American Naturalist, 108, 59–74. 10.1086/282885

[ece36099-bib-0023] Stamps, J. A. , Briffa, M. , & Biro, P. A. (2012). Unpredictable animals: Individual differences in intraindividual variability (IIV). Animal Behaviour, 83, 1325–1334. 10.1016/j.anbehav.2012.02.017

