# Supplementary material for: Intraindividual variability in behavior shapes fitness landscapes
Source: Ecol Evol. 2020 Feb 15;10(6):2838–45. doi: 10.1002/ece3.6099 (PMC7083662; doi:10.1002/ece3.6099)
Supplement: Supplementary file 5 [file ECE3-10-2838-s005.docx]

**Supporting Information**

**Movie 1** corresponds with Figs. 3 and 5 (static), **Movie 2** corresponds with Fig. 3 (stochastic), **Movie 3** corresponds with Fig. 5 (stochastic), and **Movie 4** corresponds with Fig. 6 (initial population: *µ_i_* = 25 and $\sigma_{i}^{\mathrm{IIV}}$= 0 for all *i*). In all movie files, points show the behavioral strategies of 1000 individuals. Figs 3 and 5 shows the histograms of $\sigma_{i}^{\mathrm{IIV}}$ (*i* = 1, …, 1000) at 1000^th^ generation of the corresponding movies.
